# Alcohol-induced autophagy via upregulation of PIASy promotes HCV replication in human hepatoma cells

**DOI:** 10.1038/s41419-018-0845-x

**Published:** 2018-09-05

**Authors:** Meihua Ran, Hui Chen, Bingyu Liang, Weibo Liao, Junjun Jiang, Jiegang Huang, Chuanyi Ning, Ning Zang, Bo Zhou, Yanyan Liao, Huifang Liu, Fengxiang Qin, Quanlue Yang, Jieliang Li, Wenzhe Ho, Hao Liang, Li Ye

**Affiliations:** 1 Guangxi Key Laboratory of AIDS Prevention and Treatment & Guangxi Universities Key Laboratory of Prevention and Control of Highly Prevalent Disease, School of Public Health, Guangxi Medical University, Nanning, 530021 Guangxi China; 2 Geriatrics Digestion Department of Internal Medicine, The First Affiliated Hospital of Guangxi Medical University, Nanning, 530021 Guangxi China; 3 Guangxi Collaborative Innovation Center for Biomedicine, Life Sciences Institute, Guangxi Medical University, Nanning, 530021 Guangxi China; 4 Department of Pathology and Laboratory Medicine, Temple University School of Medicine, Philadelphia, PA 19140 USA

## Abstract

Both alcohol and hepatitis C virus (HCV) infection could induce cellular autophagy in liver cells, which is considered to be essential for productive HCV replication. However, whether alcohol-induced autophagy is involved in the pathogenesis of HCV infection is still poorly understood. Alcohol treatment could induce autophagy in Huh7 cells (a hepatoma cell line that supports HCV JFH-1 replication), evidenced by the increase of LC3B-II levels, the conversion of LC3B-I to LC3B-II, and the formation of GFP-LC3 puncta as well as the decrease of p62 level in alcohol-treated cells compared with control cells. Alcohol treatment also significantly increased PIASy (a member of the PIAS family) expression, which can act as a SUMO (small ubiquitin-like modifier protein) E3 ligase to regulate a broader range of cellular processes including autophagy. Overexpression or the silencing expression of PIASy in alcohol-treated Huh7 cells could increase or decrease autophagic activation caused by alcohol treatment, respectively, and thus affect HCV replication correspondingly. In the absence of alcohol, overexpression or silencing expression of PIASy increase or decrease the level of cellular autophagy, judged by the changes of LC3B-II and p62 levels in the presence or absence of chloroquine (CQ), a lysosome inhibitor. More importantly, in the presence of 3-methyladenine (3-MA), an inhibitor in the early stage of autophagy, the effects of overexpression or silencing expression of PIASy on HCV replication were largely blocked. Furthermore, PIASy could selectively drive the accumulation of SUMO1-conjugated proteins, along with upregulation of the expression of several important autophagy factors, including ATG7 and ATG5–ATG12. In conclusion, alcohol promotes HCV replication through activation of autophagy in Huh7 cells, which partly attributes to its induction of PIASy expression. PIASy-enhanced accumulation of SUMO1-conjugated proteins may contribute to its inducing effect of autophagy. Our findings provide a novel mechanism for the action of alcohol-promoting HCV replication in the context of cellular autophagy.

## Introduction

Hepatitis C virus (HCV) infection and alcohol abuse represent the two main causes of chronic liver disease worldwide^1,2^. Currently, it is estimated that approximately 71.1 million individuals globally are living with HCV infection^3^, and chronic HCV infection may lead to cirrhosis and hepatocellular carcinoma (HCC)^4^. Alcoholic liver disease is a direct consequence of chronic alcohol consumption and is recognized as an important health problem worldwide. Chronic or acute alcohol abuse often leads to liver injury associated with alcoholic hepatitis, liver fibrosis, cirrhosis, and liver cancer^5^. Previous studies have indicated that HCV infection and alcoholism coexist in a large number of people. Alcoholic individuals have high seroprevalence of HCV infection^1^, and among patients with chronic HCV infection, heavy alcohol consumption is rather common^6,7^. HCV and alcohol most likely act synergistically to accelerate the development and progression of liver disease^5^.

The role of alcohol in promoting HCV-related liver diseases has been suggested in a number of clinical investigations. Mechanism research has revealed that alcohol and HCV may synergistically accelerate the development of liver diseases by enhancement of HCV replication, suppression of innate immunity^8,9^, increased oxidative stress^10^, generation of reactive oxygen species (ROS), iron accumulation, and steatosis induction^2,11^. These findings also imply that the interactions between alcohol and HCV are very complex and need to be further illustrated. Although the introduction of direct-acting antiviral (DAA) therapies for treatment of HCV infection has dramatically improved treatment responses and represents a milestone in the HCV treatment landscape, better understanding of the underlying mechanisms responsible for the alcohol effect on HCV infection/replication would provide new insights into their interaction, as well as information for clinical treatment and management of alcoholic patients with chronic HCV infection, which yet does not have standard guidelines for whether or how long alcohol abuse is abstinent before beginning the HCV treatment, even in the DAA era^12^.

Autophagy is predominantly a protective mechanism, acting as a cleanser to remove damaged organelles and cytosolic components^13^. However, recent studies have highlighted the close interplay of autophagy and HCV. HCV has evolved to utilize autophagy to complete its own replication, and autophagy machinery plays an important role in HCV pathogenesis^14,15^. The autophagy-related proteins, including Beclin 1, LC3, Atg4B, Atg5, Atg7, and Atg12, have been identified to be proviral factors that are important for productive HCV replication^16–20^.On the other hand, HCV has the ability to induce autophagy to enhance its replication, HCV can induce the accumulation of autophagosomes, and use autophagosomal membranes as the site for its RNA replication^20,21^. Enhancement of cellular autophagy, by either HCV infection itself or other non-HCV factors, could increase the production of HCV viral particles and favor HCV propagation^18,22^.

Autophagy also plays a pivotal role in the pathogenesis of alcohol-related liver disease^23^. A number of recent reports have shown that alcohol exposure has a significant effect on hepatic autophagy, and most of them support that alcohol can activate hepatic autophagy in vivo, in cultured primary hepatocytes, and in mice models^24–28^, except that a few studies reported impairment of liver autophagy in chronic alcohol-administered mice^29^. Our preliminary data showed that alcohol treatment could induce cellular autophagy in Huh 7 cells, a hepatoma cell line that supports the full cycle of HCV JFH-1 infection/replication^30^. Although the precise mechanism by which alcohol consumption affects hepatic autophagy remains unclear, alcohol definitely has an impact on autophagy in liver cells. In this case, it is interesting to illuminate whether the alcohol-induced (or alcohol-inhibited) autophagy plays a role in HCV replication. In this study, we examined whether alcohol enhances HCV infection/replication via regulation of cellular autophagy in human hepatoma Huh7 cells. We also explored the mechanism(s) at cellular and molecular levels involved in alcohol action on HCV replication in the context of autophagy.

## Materials and methods

### Reagents

Alcohol was purchased from Kelong Bio Inc. (Chengdu, China). Chloroquine (CQ), 3-methyladenine (3-MA), anti-P62/SQSTM1 antibody, and secondary antibodies for western blot (horseradish peroxidase-conjugated goat-anti-rabbit IgG, and goat-anti-mouse IgG) were purchased from Sigma-Aldrich China (Shanghai, China). Anti-β-actin, anti-ATG7, anti-ATG3 antibodies were purchased from Abcam Trading China (Shanghai, China). Anti-HCV core, anti-ATG5–ATG12, and Lipofectamine3000 were purchased from Thermo Fisher Scientific China (Shanghai, China). Anti-PIASy, anti-LC3B, anti-Beclin 1, anti-SUMO1, and anti-SUMO2 were purchased from Cell Signaling Technology China (Shanghai, China), and anti-CYP2E1 and anti-ADH were purchased from Novus Biologicals China (Shanghai, China).

### Cell culture, HCV JFH-1 infection, and plasmid/shRNA transfection

The human hepatoma cell line Huh7 and HCV JFH-1 virus were kindly provided by Dr. Wenzhe Ho (Temple University, Philadelphia, USA). Huh7 cells were cultured in high-glucose Dulbecco’s modified Eagle’s medium (DMEM), supplemented with 10% fetal bovine serum (FBS), 100 nM nonessential amino acids (NEAA), 100 U/mL penicillin, and 100 µg/mL streptomycin. The cells were cultured at 37 °C in a humidified incubator with an atmosphere of 5% CO_2_. Infectious HCV JFH-1 was generated by transfection of in vitro- transcribed genomic JFH-1 RNA to Huh7 cells as previously described^30^. Infection of Huh7 cells with HCV JFH-1 was carried out at a multiplicity of infection (MOI) of 0.1. PIASy overexpression or silencing expression was carried out by transfection of plasmid containing PIASy (pCMV-myc-PIASy) or shRNA against PIASy, respectively. shRNA-PIASy and control-scrambled shRNA (the sequence is not available from the supplier) were synthesized from Qiagen, China (Shanghai, China). Transfection of plasmids or shRNA to Huh7 cells was carried out using Lipofectamine 3000 from Thermo Fisher Scientific China (Shanghai, China).

### Alcohol treatment

HCV-infected or uninfected Huh7 cells in 24-well or 12-well plates were incubated with or without alcohol (20–80 mM) for up to 144 h. The alcohol concentrations choice for the study was based on our previous studies^9,31^.

### Quantitative real-time RT-PCR (RT-PCR)

Total cellular RNA was extracted from Huh7 cells using TaKaRa MiniBEST and Universal RNA Extraction Kits from Takara Company (Dalian, China). The RNA was then reversely transcribed to cDNA using the reverse transcription kit (Takara, Dalian, China), following the manufacturer’s instruction. RT-PCR was performed using SYBR Green PCR Master Mix (Takara, Dalian, China) and a StepOne Plus real-time PCR system (Life Technologies). The special oligonucleotide primers used in this study were listed in Supplementary Table 1.

### Western blot

The cell lysates were prepared using radioimmunoprecipitation assay (RIPA) buffer (MultiSciences Biotech, Hangzhou, China) plus 1% protease inhibitor cocktail (Asvio Technology, Guangzhou, China). Protein concentrations were determined by DC protein assay. Western blot assay was carried out as previously described^32^. The primary antibodies used in this study were as follows: anti-β-actin (1:2000), anti-PIASy (1:1000), anti-HCV core (1:3000), anti-LC3B (1:2000), anti-p62 (1:2000), anti-Atg7 (1:1000), anti-Atg5– Atg12 (1:1000), anti-SUMO1 (1:1000), anti-SUMO2/3 (1:1000), anti-ADH1 (1:1000), and anti-CYP2E1 (1:1000). The secondary antibodies were horseradish peroxidase-conjugated goat-anti-rabbit IgG (1:10,000) or goat-anti-mouse IgG (1:5000), respectively. The immunoreactive bands were visualized by SuperSignal West Pico chemiluminescence substrate (Thermofisher, USA). The band densities were measured by Image J software (National Institutes of Health, Bethesda, MD, USA). The values were normalized to β-actin.

### Autophagy analysis by fluorescence microscope

The Huh7 cells were transfected with lentivirus-mediated transient green fluorescent protein (GFP)-LC3 to generate GFP-LC3-expressing cells. The lentiviral vector containing GFP-LC3 fusion gene and lentivirus were purchased from Genechem Company (Shanghai, China). Autophagy was assessed using GFP-LC3 redistribution in cells that was detected by an inverted fluorescence microscope (Nikon Ti-s). The number of GFP-LC3-positive dots per cell was determined in three independent experiments.

### Statistical analysis

Data were expressed as mean ± standard deviation (SD). All assays were repeated at least three times, and each experiment was performed at least in triplicate. We determined the statistical significance between two groups by Student’s *t* test and among multiple groups by one-way analysis of variance (ANOVA). The *p* value *<* 0.05 was considered significant.

## Results

### Alcohol treatment promotes HCV JFH-1 replication in Huh7 cells

**Fig. 1:**
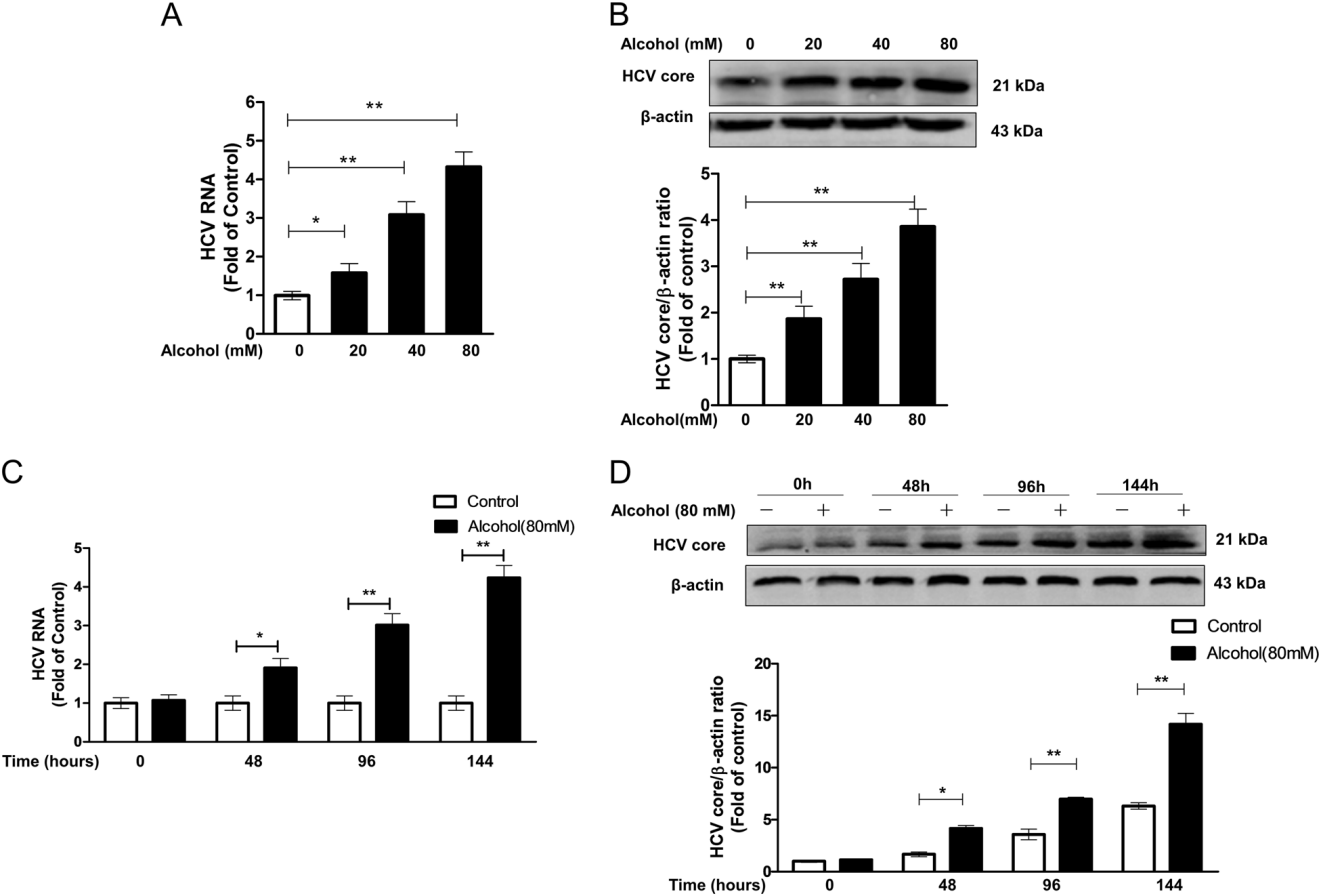
Alcohol enhances HCV replication in Huh7 cells. Huh7 cells were infected with HCV JFH-1. At day 3 post infection, the cells were treated with or without alcohol under different conditions. **a**, **b** The dose effect of alcohol on HCV replication. The cellular RNA and proteins were extracted for RT-PCR (**a**) and western blot analyses (**b**). **a** The levels of intracellular HCV RNA, with normalization to the corresponding GAPDH mRNA level, are expressed as the fold of control (without alcohol treatment, which was defined as 1). **b** A representative western blot image shows HCV core protein expression in alcohol-treated or untreated control cells. The densitometric intensities of the HCV core and β-actin bands were quantified by Image J software. The relative HCV core/β-actin ratios were calculated and shown as the fold of control (without alcohol treatment, which was defined as 1). **c**, **d** The time-course effect of alcohol on HCV replication. HCV JFH-1-infected Huh7 cells were treated with or without alcohol (80 mM) for different times (0, 48, 96, and 144 h). The cellular RNA and proteins were extracted for real-time RT-PCR (**c**) and western blot assay (**d**). **c** The levels of intracellular HCV RNA, with normalization to the corresponding GAPDH mRNA level, are expressed as the fold of control (without alcohol treatment, which was defined as 1 at different time points, respectively). **d** A representative western blot image shows HCV core protein expression in alcohol-treated or untreated control cells. The relative HCV core/β-actin ratios were calculated and shown as the fold of control (without alcohol treatment, which was defined as 1 at different time points, respectively). The data are the mean ± SD of the results of three independent experiments. *p < 0.05, **p < 0.01

We first verified whether alcohol promotes HCV replication in Huh7 cells. HCV-JFH-1-infected Huh7 cells, at day 3 post infection, were treated with alcohol at different concentrations for 96 h (Fig. 1a, b) or at 80 mM for different times (Fig. 1c, d). The alcohol concentrations and treatment time selected for the study were based on our previous studies^9,31^, which indicated that alcohol at the concentration of 100 mM or lower had no cytotoxic effect on Huh7 cells. As shown in Fig. 1, when HCV JFH-1-infected Huh7 cells were treated with alcohol, the HCV RNA level was significantly increased in a dose-dependent and time-dependent manner (Fig. 1a, c), which was also confirmed at HCV core protein level by western blot assay (Fig. 1b, d). Therefore, we concluded that alcohol could promote HCV JFH-1 replication in Huh7 cells. In addition, several studies indicated that the effects of alcohol on HCV replication in Huh7 or Huh7-derived cells (Huh7.5 and Huh7.5.1) are involved in alcohol metabolism, and thus the expression of the two main alcohol-metabolizing enzymes, alcohol dehydrogenase (ADH) and cytochrome P450-2E1 (CYP2E1) in Huh7 cells is important for the action of alcohol on HCV. We used western blot to detect the expression of these two enzymes in Huh7 cells and found that, although Huh7 cells express low levels of ADH and CYP2E1, when the cells were treated with alcohol, the expression of these two enzymes was induced to relatively high levels (Supplementary Fig. 1).

### Alcohol treatment induces cellular autophagy in Huh7 cells

**Fig. 2:**
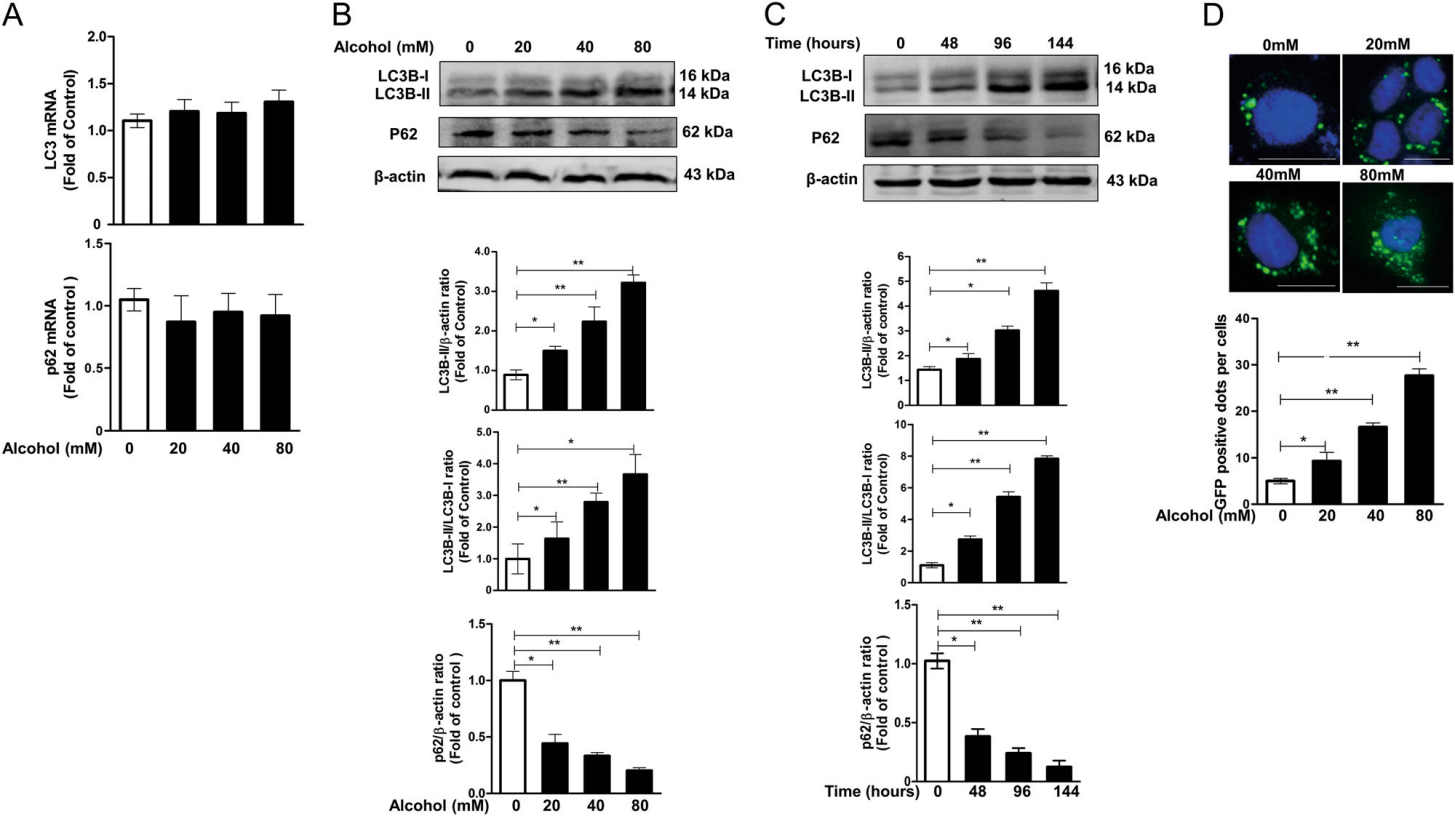
The effect of alcohol on the levels of two autophagy markers, LC3B and p62, in Huh7 cells. **a** The effect of alcohol on mRNA expression of LC3 and p62. Huh7 cells were incubated with or without alcohol at indicated concentrations for 96 h. The levels of LC3 and p62 mRNA, with normalization to the corresponding GAPDH mRNA level, are expressed as the fold of control (without alcohol treatment, which was defined as 1). **b** The dose effect of alcohol on protein levels of LC3B and p62. Huh7 cells were treated with or without alcohol at indicated concentrations for 96 h. A representative western blot image shows the protein levels of LC3B-I, LC3B-II, and p62 in alcohol-treated or untreated control cells. The densitometric intensities of western blot bands were quantified by Image J software. The relative ratios LC3B-II/β-actin, LC3B-II/LC3B-I, and p62/β-actin were calculated and shown as the fold of control (without alcohol treatment, which was defined as 1). **c** The time-course effect of alcohol on protein levels of LC3B and p62. Huh7 cells were treated with or without alcohol (80 mM) for different times (0, 48, 96, and 144 h). A representative western blot image shows the protein levels of LC3B-I, LC3B-II, and p62. The relative ratios of LC3B-II/β-actin, LC3B-II/LC3B-I, and p62/β-actin were calculated and shown as the fold of control (without alcohol treatment, which was defined as 1). **d** Huh7 cells expressing GFP-LC3 protein were treated with alcohol at indicated concentrations for 96 h. GFP-LC3-positive dots per cell were determined by fluorescence microscope (30 cells were counted per experiment). Scale bar: 50 µm. The data are the mean ± SD of the results of three independent experiments. *p < 0.05, **p < 0.01

To examine whether alcohol affects cellular autophagy in Huh7 cells, we first investigated whether alcohol modulates the expression of two markers of autophagy, microtubule-associated protein 1 light chain 3 (LC3) and p62. LC3, a cytosolic ubiquitin-like protein, has two forms, LC3-I and LC3-II. During autophagy activation, the soluble form of LC3 (LC3-I) is converted into a lipidated form (LC3-II), which is associated with autophagosomal membranes. p62, additionally a measure of autophagic flux, is a cargo receptor for autophagic degradation of ubiquitinated targets that binds directly to LC3-II, and is exclusively degraded during autophagy. The decreased levels of p62 could be observed when autophagy is activated. Thus, the relative levels of LC3B-II and p62 were used as the markers of autophagy and were always first analyzed in autophagy. In this study, at the mRNA level, no significant difference (*p* > 0.05) was observed in LC3 or p62 expression between alcohol-treated cells and alcohol-untreated cells (Fig. 2a). At protein level, however, significantly increased levels of LC3B-II protein (LC3B-II/β-actin) and conversion of LC3B-I into LC3B-II (LC3B-II/LC3B-I) were observed in alcohol-treated cells compared with control cells, and the increase was in a dose- and time-dependent manner (Fig. 2b, c). Meanwhile, the levels of p62 (p62/β-actin) decreased along with the increasing levels of LC3B-II protein, either in a dose- or time-dependent manner (Fig. 2b, c). Huh7 cells expressing GFP-LC3 were also used to examine the effect of alcohol on the autophagic activity that was indicated by the numbers of GFP-LC3 dots (Fig. 2d). A significant impact of alcohol on GFP-LC3 dots was observed. The numbers of GFP-LC3 dots in alcohol-treated cells were significantly higher than that in control cells, and the effect was obvious in a dose-dependent manner (Fig. 2d).

**Fig. 3:**
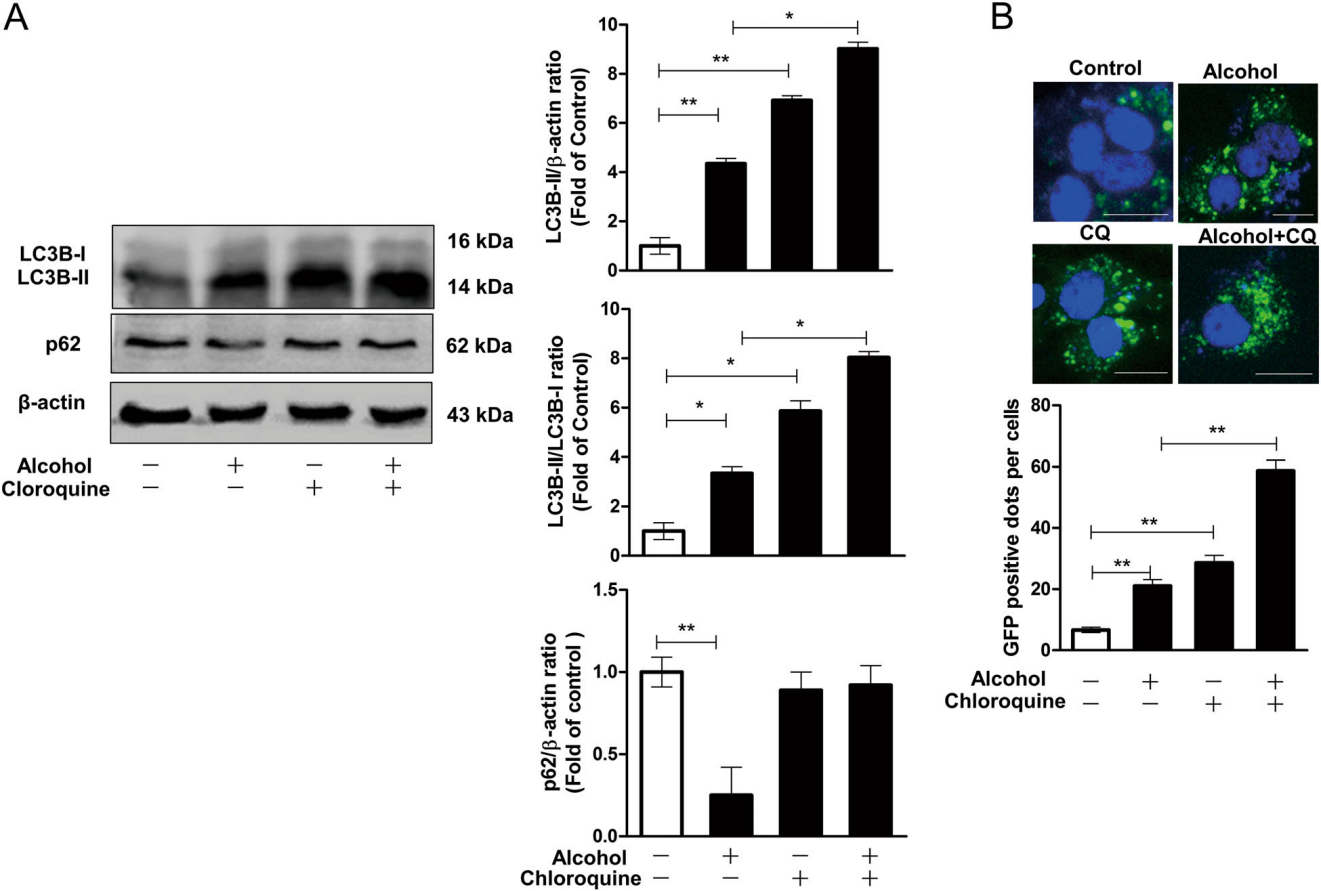
Alcohol induces autophagy flux in Huh7 cells. In the presence or absence of an autophagosome–lysosome fusion inhibitor, chloroquine (CQ, 25 uM), Huh7 cells (**a**), or Huh7 cells expressing GFP-LC3 (**b**) were treated with or without alcohol (80 mM) for 96 h. **a** A representative western blot image shows the protein levels of LC3B-I, LC3B-II, and p62 in cells. The densitometric intensities of western blot bands were quantified by Image J software. The relative ratios of LC3B-II/β-actin, LC3B-II/LC3B-I, and p62/β-actin were calculated and shown as the fold of control (without alcohol and CQ treatment, which was defined as 1). **b** GFP-LC3-positive dots per cell were determined by fluorescence microscope (30 cells were counted per experiment). Scale bar: 50 um. The data are the mean ± SD of the results of three independent experiments. *p < 0.05, **p < 0.01

Although the decrease in p62 level does reflect the induction of autophagy, the increase in LC3B-II level could be caused by either induction of autophagosome formation or inhibition of degradation in lysosomes. An autophagosome–lysosome fusion inhibitor, CQ was also used to further clarify whether the increase of LC3B-II levels caused by alcohol was due to the increased autophagosome formation or the decreased autophagosome degradation. The data revealed that the combined use of alcohol and CQ could lead to higher induced LC3B-II level compared with alcohol alone or CQ alone (Fig. 3a–c). However, the CQ treatment had little effect on p62 level because it inhibits the degradation function of lysosomes, and CQ co-treatment could also prevent the degradation of p62 caused by alcohol treatment, which was shown in the alcohol-alone group (Fig. 3a, b). The above results indicated that alcohol could induce cellular autophagy flux in Huh7 cells (Figs. 2 and 3).

### Alcohol induces PIASy expression in Huh7 cells

**Fig. 4:**
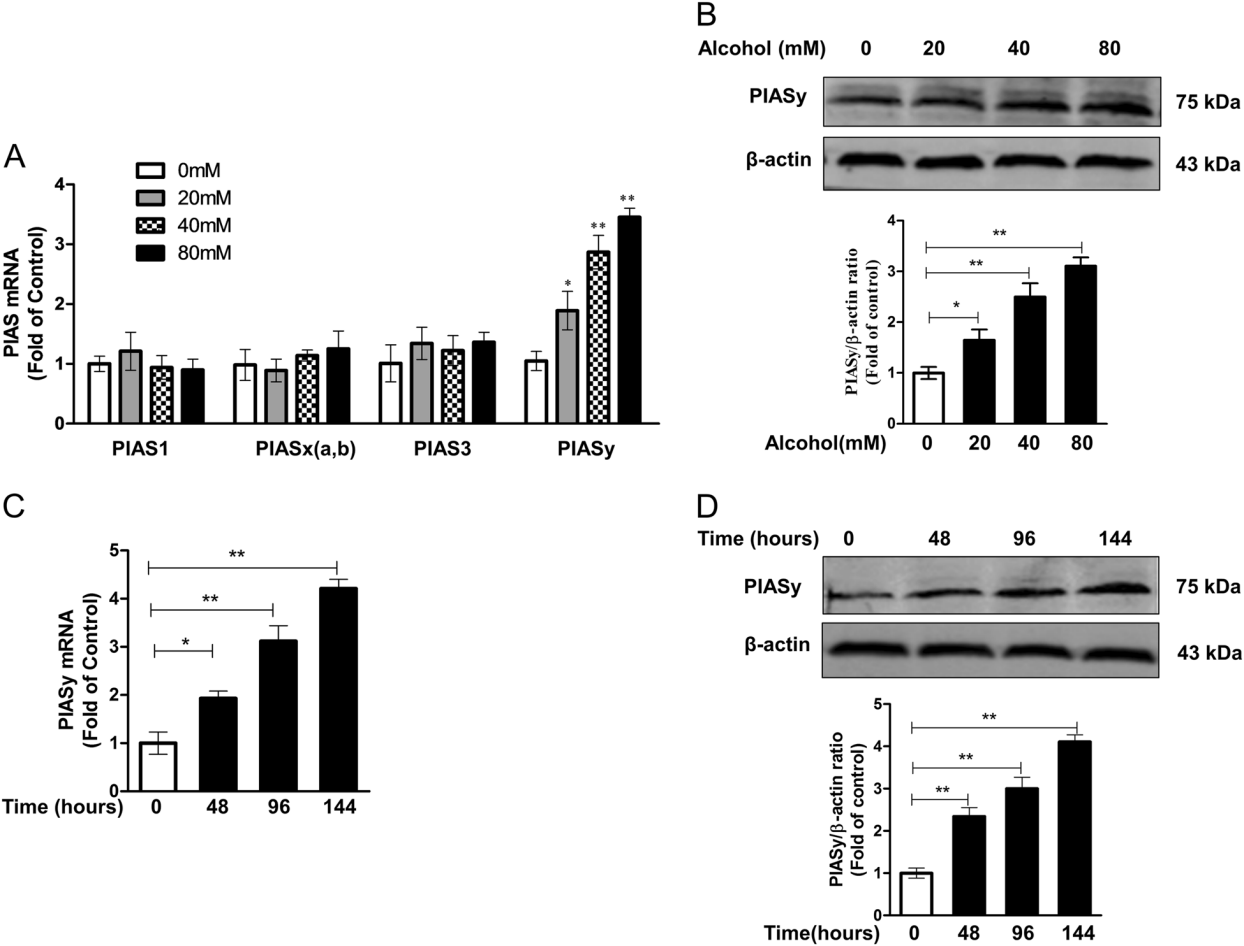
Alcohol treatment promotes PIASy expression in Huh7 cells. **a** The effect of alcohol on mRNA expression of four members (PIAS1, PIASx(a,b), PIAS3, and PIASy). Huh7 cells were treated with or without alcohol at indicated concentrations for 96 h. The mRNA levels of four types of PIASs, with normalization to the corresponding GAPDH mRNA level, are expressed as the fold of control (without alcohol treatment, which was defined as 1). **b** A representative western blot image shows the protein levels of PIASy in alcohol-treated or alcohol-untreated cells. The relative ratios of PIASy/β-actin were calculated and shown as the fold of control (without alcohol treatment, which was defined as 1). **c**, **d** Time-course effect of alcohol on PIASy expression. Huh7 cells were treated with alcohol (80 mM) for different times (0, 48, 96, and 144 h). **c** The mRNA levels of PIASy, with normalization to the corresponding GAPDH mRNA level, are expressed as the fold of control (without alcohol treatment, which was defined as 1). **d** A representative western blot image shows the protein levels of PIASy. The relative PIASy/β-actin ratios were calculated and shown as the fold of control (without alcohol treatment, which was defined as 1). The data are the mean ± SD of the results of three independent experiments. *p < 0.05, **p *<* 0.01

We then investigated whether alcohol affects the expression of the PIAS family, including PIAS1, PIASx(a,b), PIAS3, and PIASy. At the mRNA level, alcohol treatment significantly upregulated PISAy expression in a dose-dependent and time-dependent manner (Fig. 4a, c). However, no significant effect (*p* > 0.05) was observed in the expression of other PIASs (PIAS1, PIASx(a.b), and PIAS3) (Fig. 4a). Western blot assay confirmed that alcohol could induce PIASy expression in Huh7 cells in a dose-dependent and time-dependent manner (Fig. 4b, d).

### Alcohol-induced PIASy is involved in alcohol-enhanced HCV replication and alcohol-induced autophagy

**Fig. 5:**
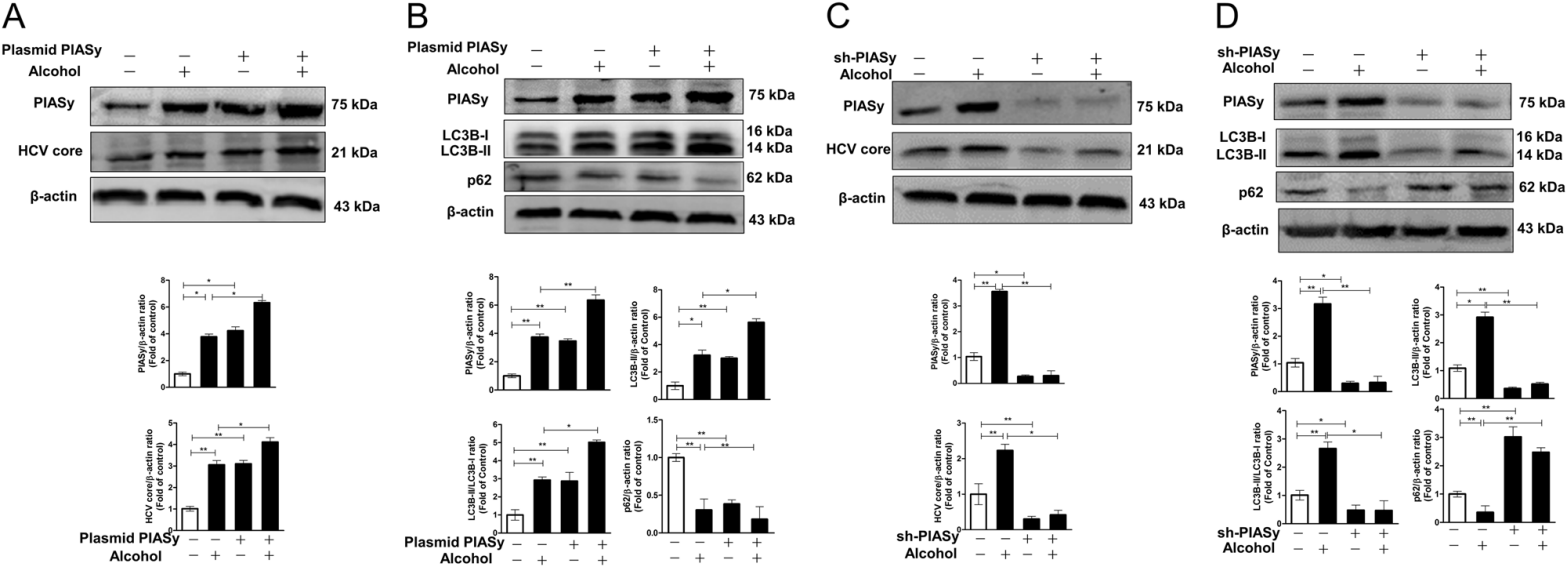
PIASy is involved in alcohol-enhanced HCV replication and alcohol-induced autophagy. **a**, **b** The effect of overexpression of PIASy on alcohol-enhanced HCV replication and alcohol-induced autophagy. **a** HCV JFH-1-infected Huh 7 cells were transfected with pCMV-myc-PIASy or empty vector. At 12 h post transfection, the cells were treated with or without alcohol (80 mM) for 72 h. The cellular proteins were extracted for western blot assay. The relative ratios of PIASy/β-actin and HCV core/β-actin were calculated and shown as the fold of control (without alcohol treatment, which was defined as 1). **b** Huh 7 cells were transfected with pCMV-myc-PIASy or empty vector. At 12 h post transfection, the cells were treated with or without alcohol (80 mM) for 72 h. The cellular proteins were extracted for western blot assay. The relative ratios of PIASy/β-actin, LC3B-II/β-actin, LC3B-II/LC3B-I, and p62/β-actin were calculated and shown as the fold of control (without alcohol treatment, which was defined as 1). **c**, **d** The effect of silencing expression of PIASy on alcohol-enhanced HCV replication and alcohol-induced autophagy. **c** HCV JFH-1-infected Huh 7 cells were transfected with shRNA-PIASy or control-scrambled shRNA. At 12 h post transfection, the cells were treated with or without alcohol (80 mM) for 72 h. The cellular proteins were extracted for western blot assay. The relative ratios of PIASy/β-actin and HCV core/β-actin were calculated and shown as the fold of control (without alcohol treatment, which was defined as 1). **d** Huh 7 cells were transfected with shRNA-PIASy or control-scrambled shRNA. At 12 h post transfection, the cells were treated with or without alcohol (80 mM) for 72 h. The cellular proteins were extracted for western blot assay. The relative ratios of PIASy/β-actin, LC3B-II/β-actin, LC3B-II/LC3B-I, and p62/β-actin were calculated and shown as the fold of control (without alcohol treatment, which was defined as 1). The data are the mean ± SD of the results of three independent experiments. *p *<* 0.05, **p < 0.01

To investigate whether alcohol-induced PIASy plays a role in alcohol-enhanced HCV replication and alcohol-induced autophagy, overexpression (pCMV-myc-PIASy) or silencing expression (shRNA-PIASy) of PIASy experiments were performed. Overexpression of PIASy could promote HCV replication and further enhance the promoting effect of alcohol on HCV replication, evidenced by increased HCV core protein level in PIASy-transfected cells compared with control cells (Fig. 5a). Silencing expression of PIASy, on the contrary, inhibited HCV replication and weakened the alcohol-enhanced HCV replication (Fig. 5c). Similarly, overexpression or silencing expression of PIASy could enhance or weaken alcohol-induced autophagy, respectively, evidenced by the increased levels of LC3B-II (LC3B-II/β-actin and LC3B-II/LC3B-I) and decreased levels of p62 (p62/β-actin) in overexpression groups and opposite changes in silencing groups (Fig. 5b, d).

### The effects of PIASy on the autophagy flux in Huh7 cells

**Fig. 6:**
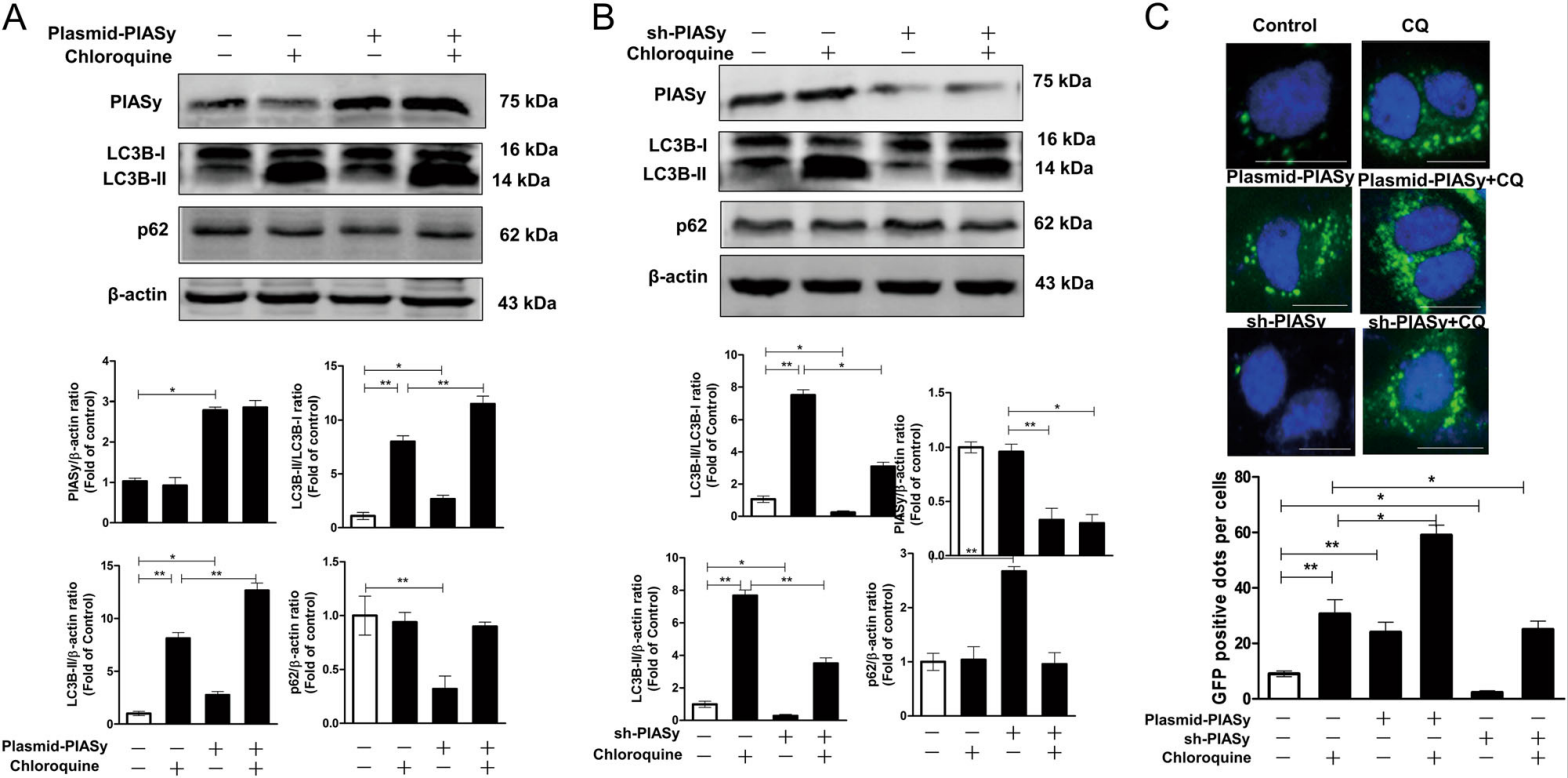
PIASy induces autophagpy flux in Huh7 cells. In the presence or absence of an autophagosome–lysosome fusion inhibitor, chloroquine (CQ, 25 uM), Huh7 cells (**a**, **b**), or Huh7 cells expressing GFP-LC3 (**c**) were transfected with pCMV-myc-PIASy/empty vector (**a**, **c**) or shRNA-PIASy/control-scrambled shRNA (**b**, **c**) for 96 h. **a** A representative western blot image shows the protein levels of PIASy, LC3B-I, LC3B-II, and p62 in PIASy-overexpressed or control cells. The relative ratios of PIASy/β-actin, LC3B-II/β-actin, LC3B-II/LC3B-I, and p62/β-actin were calculated and shown as the fold of control (without CQ treatment and with empty vector transfection, which was defined as 1). **b** A representative western blot image shows the protein levels of PIASy, LC3B-I, LC3B-II, and p62 in PIASy-down-expressed or control cells. The relative ratios of PIASy/β-actin, LC3B-II/β-actin, LC3B-II/LC3B-I, and p62/β-actin were calculated and shown as the fold of control (without CQ treatment and with scrambled shRNA transfection, which was defined as 1). **c** GFP-LC3-positive dots per cell were determined by fluorescence microscope (30 cells were counted per experiment) in PIASy-overexpressed, PIASy-down-expressed, or control cells. Scale bar: 50 µm. The data are the mean ± SD of the results of three independent experiments. *p < 0.05, **p *<* 0.01

Since alcohol-induced PIASy had a significant effect on cellular autophagy, next, we examined whether PIASy itself affects autophagy via overexpression or silencing expression of PIASy in Huh7 cells, in the presence or absence of CQ. In the absence of CQ, the increased LC3B-II levels (LC3B-II/β-actin, LC3B-II/LC3B-I, or GFP-LC3 dots) and decreased p62 levels (p62/β-actin) were observed in pCMV-myc-PIASy-transfected cells compared with control cells (Fig. 6a, c), whereas the opposite changes of LC3B-II and p62 levels were observed in shRNA-PIASy-transfected cells (Fig. 6b, c). In the presence of CQ, the overexpression or silencing expression of PIASy could enhance or decrease the level of LC3B-II, respectively, which was accumulated by CQ inhibition of degradation in lysosomes (Fig. 6a–c); however, the p62 levels were not affected by overexpression or silencing expression of PIASy (Fig. 6a, b), also due to the inhibitory effect of CQ on lysosomes. These results indicated that PIASy itself could activate autophagy in Huh7 cells.

### PIASy promotes HCV replication via activation of autophagy in Huh7 cells

**Fig. 7:**
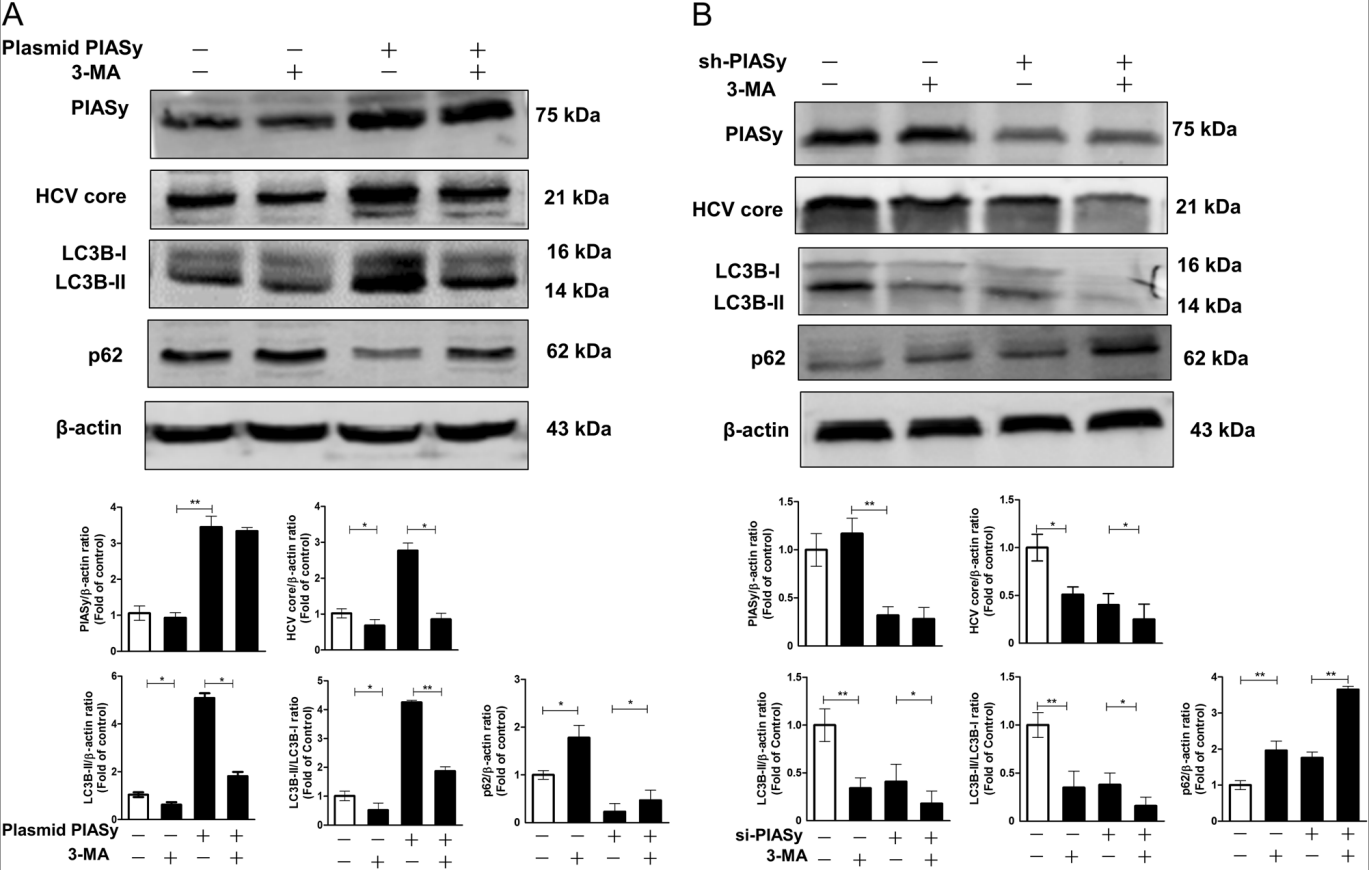
The effects of the inhibition of PIASy-induced autophagy on HCV replication in Huh7 cells. In the presence or absence of an inhibitor in the early stage of autophagy, 3-methyladenine (3-MA,10 mM), HCV JFH-1-infected Huh7 cells were transfected with pCMV-myc-PIASy/empty vector (**a**) or shRNA-PIASy/control-scrambled shRNA (**b**) for 96 h. **a** A representative western blot image shows the protein levels of PIASy, HCV core, LC3B-I, LC3B-II, and p62 in PIASy-overexpressed or control cells. The relative ratios of PIASy/β-actin, HCV core/β-actin, LC3B-II/β-actin, LC3B-II/LC3B-I, and p62/β-actin were calculated and shown as the fold of control (without 3-MA treatment and with empty vector transfection, which was defined as 1). **b** A representative western blot image shows the protein levels of PIASy, HCV core, LC3B-I, LC3B-II, and p62 in PIASy-down-expressed or control cells. The relative ratios of PIASy/β-actin, HCV core/β-actin, LC3B-II/β-actin, LC3B-II/LC3B-I, and p62/β-actin were calculated and shown as the fold of control (without 3-MA treatment and with scrambled shRNA transfection, which was defined as 1). The data are the mean ± SD of the results of three independent experiments. *p < 0.05, **p < 0.01

Next, we investigated whether PIASy-induced autophagy contributes to HCV replication in Huh7 cells in the presence or absence of 3-methyladenine (3-MA), an inhibitor in the early stage of autophagy that inhibits conversion of LC3-I into LC3-II. As shown in Fig. 7, 3-MA treatment efficiently led to decreased levels of LC3B-II and suppression of autophagy (Fig. 7a, b). Correspondingly, HCV core protein levels were also downregulated by 3-MA treatment (Fig. 7a, b), indicating that inhibition of autophagy could suppress HCV replication. In the absence of 3-MA, overexpression of PIASy greatly promoted HCV replication along with induction of autophagy (Fig. 7a), whereas the silencing expression of PIASy weakened HCV replication along with less-activated autophagy observed (Fig. 7b). However, in the presence of 3-MA, since the cellular autophagic flux was inhibited, the enhancing effect of PIASy overexpression or the inhibitory effect of silencing expression on HCV replication were not observed as those in the absence of 3-MA (Fig. 7a, b). These results indicated that PIASy promotes HCV replication via activation of autophagy.

### PIASy induces autophagy through inducing the accumulation of SUMO1-conjugated proteins

**Fig. 8:**
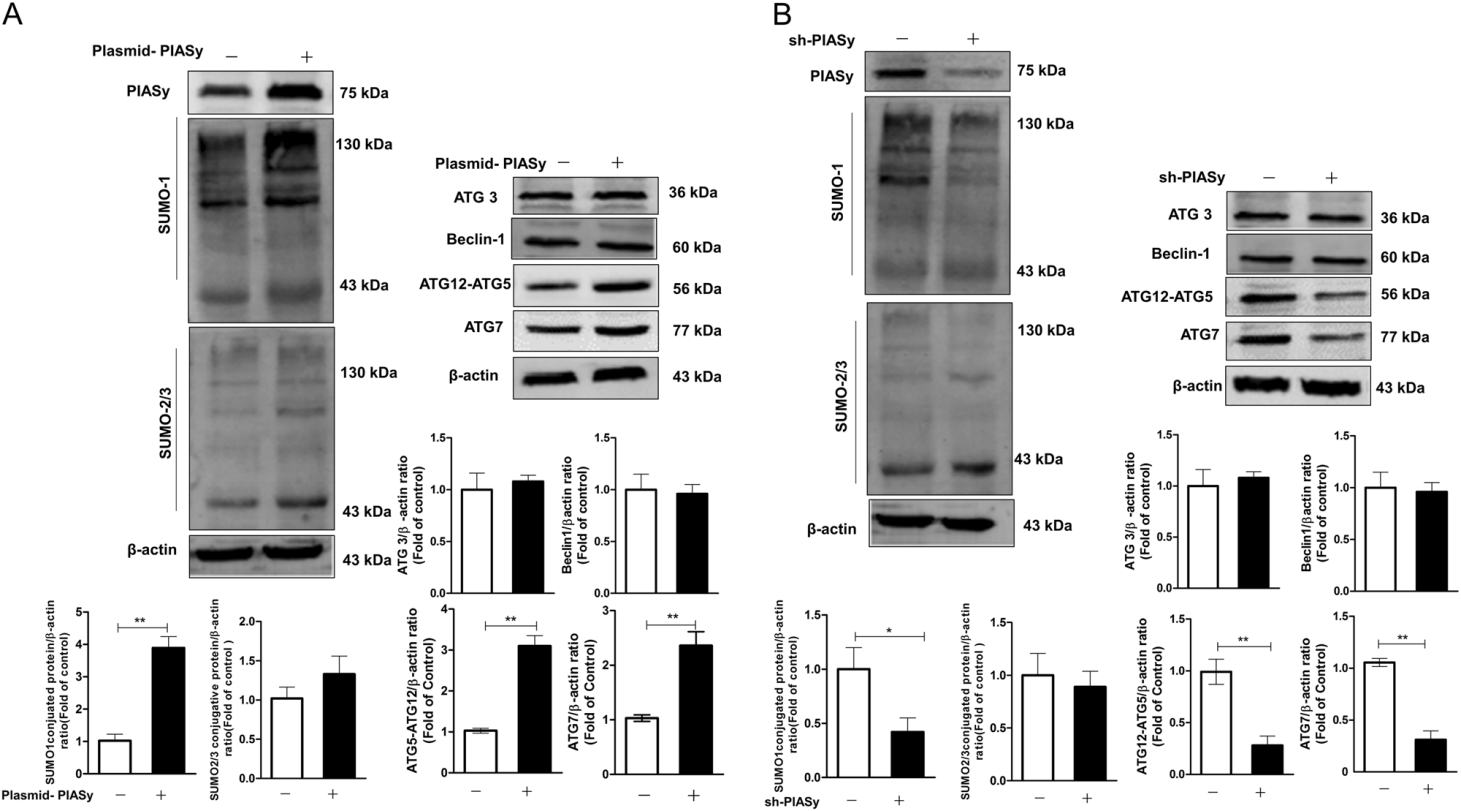
PIASy induces autophagy via regulation of SUMO1 pathway in Huh7 cells. The effect of overexpression (**a**) or silencing expression (**b**) of PIASy on levels of SUMO-conjugated proteins and autophagy-related genes. Huh7 cells were transfected with pCMV-myc-PIASy/empty vector (**a**) or shRNA-PIASy/control- scrambled shRNA (**b**) for 96 h. The western blot images show the protein levels of PIASy, SUMO1-conjugated proteins, SUMO2/3-conjugated proteins, Atg7, and Atg5–12 in PIASy-overexpressed (**a**) or PIASy-downexpressed (**b**) cells. The relative ratios of PIASy/β-actin, Atg7/β-actin, and Atg5–12/LC3B-I were calculated and shown as the fold of control (with empty vector transfection (**a**) or with scrambled shRNA transfection (**b**), which was defined as 1, respectively). The data are the mean ± SD of the results of three independent experiments. *p < 0.05, **p < 0.01

PIASy belongs to a small ubiquitin-like modifier (SUMO) E3 ligase^33^, and SUMO proteins could modify several components of autophagy to regulate cellular autophagy^34,35^. To assess whether PIASy induces autophagy through the SUMO pathway, we investigate the effect of PIASy on SUMO expression via overexpression or silencing expression of PIASy in Huh7 cells. As shown in Fig. 8, overexpression of PIASy stimulated the expression of SUMO1-conjugated proteins, however, it had little effect on SUMO2/3-conjugated proteins (Fig. 8a). On the contrary, silencing PIASy expression led to decreased expression of SUMO1-conjugated proteins (Fig. 8b), and had little effect on SUMO2/3-conjugated proteins. Since SUMO1- conjugated proteins have a close connection with autophagy, we examined whether the essential and important autophagy-related genes (ATG) were affected by PIASy expression along with the levels of SUMO1-conjugated proteins. Overexpression of PIASy led to increased levels of ATG7 and ATG5–ATG12, but had little effect on ATG3 and Beclin-1 expression (Fig. 8a). Silencing expression of PIASy resulted in decreased levels of ATG7 and ATG5–ATG12, and had little effect on ATG3 and Beclin-1 expression (Fig. 8b).

## Discussion

In the liver, autophagy is an essential mechanism that maintains the balance of energy and nutrients, cellular maintenance, and hepatic function^36^, and it protects the liver from various injuries, including alcohol-induced oxidative stress and accumulation of lipid droplets^24,37^. So far, a number of studies have investigated the effects of alcohol exposure on hepatic autophagy, and most of them indicated that alcohol activates hepatic autophagy^24–28^, but few studies reported that alcohol consumption led to impairment of liver autophagy^29^. Consistent with the results of most of previous studies, we observed that alcohol treatment induces cellular autophagy in Huh7 cells (Figs. 2, 3). Many studies have also shown that autophagy response is induced after HCV infection and HCV hijacks the autophagy machinery to complete infection and replication^14,15^. Therefore, we considered that alcohol-induced autophagy may play a role in the enhancement of HCV replication in liver cells. However, it is hard to illustrate this issue because both alcohol and HCV infection could induce autophagy in liver cells. Previous studies indicated that alcohol-induced oxidative stress or ER (endoplasmic reticulum) stress, both of which are known to be linked to autophagy activation^38^, are also involved in HCV-induced autophagy^39,40^. Thus, we expected that we could find some new intermediate host factors that are involved in alcohol-induced autophagy but do not derive from HCV infection, to explore the role of alcohol-induced autophagy in HCV replication and the mechanism(s) involved.

Our preliminary data indicated that alcohol treatment could induce PIASy expression in Huh7 cells, which was confirmed in the present study (Fig. 4). PIASy is a member of PIAS protein family^41^. PIAS represents a family of proteins originally identified as an interaction partner with cytokine-induced STAT, which are negative regulators of innate immunity by repression of key regulators in the interferon (IFN) pathway^41–43^. In addition, PIAS is also known as SUMO E3 ligases^33^, which can serve as a scaffold that brings together SUMO-charged E2 and the substrate, thereby promoting the efficiency and specificity of the sumoylation process. SUMO (small ubiquitin-like modifier protein) is a kind of key post-translational modification that critically regulates several important cellular functions, including transcription, cell division, protein stability and translocation, signal transduction, protein–protein interactions, and chromatin segregation^44,45^. Recently, several studies have revealed that SUMO proteins play a role in the autophagy pathway. SUMO proteins could modify several components of autophagy to regulate cellular autophagy^34,35^, and SUMO protein itself may undergo autophagosomal degradation^46^. SUMO1 promotes amyloid-β production via the modulation of autophagy in Alzheimer's disease^47^. Increased UBC9-mediated SUMOylation is sufficient to induce relatively high levels of autophagy to protect the heart against a proteotoxic environment in cardiac disease^48^. Autophagy induced by elevated levels of SUMO1 impairs synaptic transmission, significantly reducing dendritic spine density and causing memory loss^49^. One recent study reported that PIASy-mediated Tip60 sumoylation regulates p53-induced autophagy^50^. These suggestive data and our preliminary results prompted us to explore the possible linkage between PIASy, SUMO, and autophagy in the process of alcohol-promoting HCV replication.

We examined whether alcohol-induced PIASy plays a role in cellular autophagy and HCV replication. Silencing expression of alcohol-induced PIASy could decrease the level of autophagy and weaken HCV replication (Fig. 5). More importantly, in the absence of alcohol treatment, PIASy itself possesses the ability to induce autophagy (Figs. 5, 6, and 7). These data provide direct evidence that PIASy is involved in alcohol-induced autophagy and alcohol-enhanced HCV replication. However, when the cellular autophagy was inhibited by the early-stage inhibitor 3-MA, the enhancement effect of HCV replication by PIASy was not completely eliminated (Fig. 7a), whereas PIASy-induced autophagy was almost inhibited, indicating that PIASy may promote HCV replication via other mechanisms. Not surprisingly, PIASy is also a negative regulator of innate immunity by repression of key regulators in the interferon (IFN) pathway^41^, which is always activated by HCV infection to limit the viral replication^51,52^. In addition, autophagy factors such as ATG5 could directly impact the innate immune pathway, which could negatively regulate type-I IFN pathway and pathogen-associated molecular pattern (PAMP)-mediated cytoplasmic retinoic acid-inducible gene I (RIG-I) pathway^15^, and thus facilitates HCV replication. Therefore, we speculated that the inhibition of the IFN pathway by PIASy, to some extent, compensates the decreased inhibitory effect of ATG5 on the IFN pathway and still enhances HCV replication under the condition of 3-MA inhibition of autophagy. Nevertheless, because the present study focused on the relationship between PIASy and autophagy, the involvement of the IFN pathway needs further study to illuminate.

In this study, we also observed that PIASy could selectively activate SUMO1 pathway, but not SUMO2/3 pathway (Fig. 8). Overexpression or silencing expression of PIASy could increase or decrease the accumulation of proteins modified by SUMO1, respectively, which is consistent with the previous study^53^. This SUMOylation was also accompanied by increased autophagy formation: increased accumulation of LC3B-II (Fig. 6c) and the formation of GFP-LC3-positive dots (Fig. 6), and the decreased level of p62 expression, which is consistent with some previous studies^47,50^ and implies that increased SUMOylation by PIASy may be a regulator to regulate autophagic flux. In addition, several important autophagy factors, including autophagy-related gene 7 (ATG7) and ATG5–ATG12 were significantly induced along with PIASy promoting SUMO1-mediated protein conjugation (Fig. 8). ATG7 and ATG12–ATG5 are key regulators of the autophagic process and play an important role in autophagosome formation. ATG7 plays a role in the early complex of autophagy and the formation of autophagic vacuoles through combining LC3 protein^22^. ATG5 forms a conjugate with ATG12 and 16L1 (ATG16), but the monomeric forms of these two proteins have been shown to be nearly undetectable under normal conditions^54^. The ATG5–ATG12/16L1 complex acts as an E3 enzyme that can promote the formation of autophagic vacuoles and promotes LC3 to be present in autophagic vacuoles^55,56^. Furthermore, direct interaction is transiently linked between ATG5 and HCV NS5B (RNA-dependent RNA polymerase) protein at a very early stage of HCV infection to facilitate the onset of viral replication^17^. In addition, the ATG5–ATG12 conjugate negatively regulates the type-I IFN production pathway by direct association with the RIG-I and IFN-beta promoter stimulator 1 (IPS-1) through the caspase recruitment domains^57^. And, HCV-exploited ATG5 negatively affects cellular immune function–type-I IFN pathway and PAMP-mediated RIG-I signaling^15^.

Taken together, in this study, we presented evidence to show that alcohol promotes HCV replication through activation of autophagy in Huh7 cells. The alcohol-activated autophagy partly attributes to its induction of PIASy expression and thus facilities HCV replication. Our findings provide a novel mechanism for the action of alcohol-promoting HCV replication in the context of cellular autophagy, which may provide some clues for novel therapeutic strategies to improve anti-HCV effects among alcohol-abused HCV-infected individuals.

## Electronic supplementary material

The primer sequences used for RT-PCR in this study

Alcohol induces the expression of alcohol dehydrogenase (ADH) and cytochrome P450-2E1 (CYP2E1) in Huh7 cells

supplementary figure legends
