# Supplementary material for: Alcohol-induced autophagy via upregulation of PIASy promotes HCV replication in human hepatoma cells
Source: Cell Death Dis. 2018 Sep 5;9(9):898. doi: 10.1038/s41419-018-0845-x (PMC6123814; doi:10.1038/s41419-018-0845-x)
Supplement: Supplementary file 1 — The primer sequences used for RT-PCR in this study [file 41419_2018_845_MOESM1_ESM.docx]

Supplementary Table 1 The primer sequences used for RT-PCR in this study

| Gene | orientation | The sequence of primers | |
| --- | --- | --- | --- |
| GAPDH | Sense | | 5’-GGTGGTCTCCTCTGACTTCAACA-3’ |
|  | Antisense | | 5’-GTTGCTGTAGCCAAATTCGTTGT-3’ |
| HCV | Sense | | 5’-RAYCACTCCCCTGTGAGGAAC-3’ |
|  | Antisense | | 5’-TGRTGCACGGTCTACGAGACCTC-3’ |
| PIAS1 | Sense | | 5’-TCCCACCCAATCTTTGTGTG-3’ |
|  | Antisense | | 5’-GCCGCATTTTACCAAGTGGA-3’ |
| PIASxa,xb | Sense | | 5’-TCTTCTGACGAAGAGGAAGACC-3’ |
|  | Antisense | | 5’-TCAGAAGATGTTCCAAGCTTCA-3’ |
| PIAS3 | Sense | | 5’-TGCTGGCCGGAACAAGAGTG-3’ |
|  | Antisense | | 5’-AGGGGGCAAAGAGAGAAGGG-3’ |
| PIASy | Sense | | 5’-GAG AAGCCCACCTGGA-3’ |
|  | Antisense | | 5’-ACACTCGCTCAGGATCTT CG-3’ |
| LC3 | Sense | | 5’- AGCAGCATCCAACCAAAATC-3’ |
|  | Antisense | | 5’-CTGTGTCCGTTCACCAACAG-3 |
| P62 | Sense | | 5’-GTGGGACAGCCAGAGGAACA-3’ |
|  | Antisense | | 5’-GCCCTTCCGATTCTGGCAT -3’ |
