# Supplementary figures and images for: Alcohol-induced autophagy via upregulation of PIASy promotes HCV replication in human hepatoma cells
Source: Cell Death Dis. 2018 Sep 5;9(9):898. doi: 10.1038/s41419-018-0845-x (PMC6123814; doi:10.1038/s41419-018-0845-x)

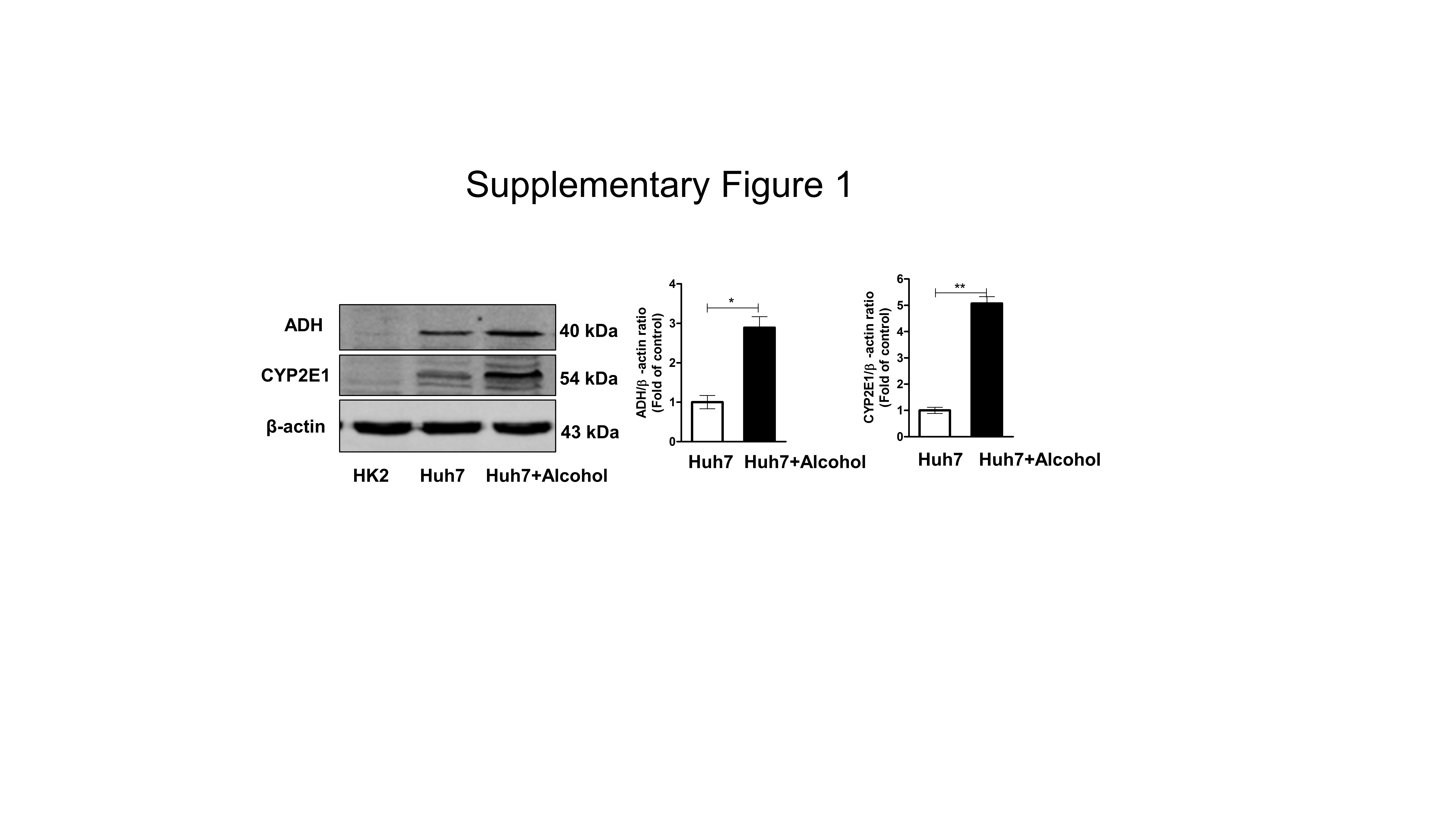

Supplement: Supplementary file 2 — Alcohol induces the expression of alcohol dehydrogenase (ADH) and cytochrome P450-2E1 (CYP2E1) in Huh7 cells [file 41419_2018_845_MOESM2_ESM.tif]
