# Supplementary material for: Alcohol-induced autophagy via upregulation of PIASy promotes HCV replication in human hepatoma cells
Source: Cell Death Dis. 2018 Sep 5;9(9):898. doi: 10.1038/s41419-018-0845-x (PMC6123814; doi:10.1038/s41419-018-0845-x)
Supplement: Supplementary file 3 — supplementary figure legends [file 41419_2018_845_MOESM3_ESM.docx]

Supplementary Figure 1. Alcohol induces the expression of alcohol dehydrogenase (ADH) and cytochrome P450-2E1 (CYP2E1) in Huh7 cells.

Huh7 cells were treated with or without alcohol (80mM) for 96h. Western blot assay shows ADH and CYP2E1 levels in HK2 cells (a negative control), alcohol-treated or alcohol-untreated Huh7 cells. The densitometric intensities of ADH, CYP2E1 and β-actin bands were quantified by Image J software. The relative ratios of ADH/β-actin and CYP2E1 complex/β-actin were calculated and shown as the fold of control (Huh7 cells without alcohol treatment, which was defined as 1). The data are the mean ± SD of the results of three independent experiments. *, *p* <0.05, **, *p* <0.01.
